# Neurological rehabilitation for a patient with chronic Rasmussen encephalitis: a case report

**DOI:** 10.1590/1980-57642021dn15-030015

**Published:** 2021

**Authors:** Nariana Mattos Figueiredo Sousa, Aidê Mascarenhas Ribeiro, Daniela Lino de Macedo Nunes

**Affiliations:** 1 Neurological Rehabilitation Program, Rede SARAH de Hospitais de Reabilitação – Salvador, BA, Brazil.

**Keywords:** encephalitis, neurological rehabilitation chronic disease, patient care team, encefalite, reabilitação neurológica, doença crônica, equipe de assistência ao paciente

## Abstract

Rasmussen encephalitis is a rare disease consisting of a chronic, slowly progressive inflammatory reaction of brain tissues. The objective of this study was to describe the case of an 18-year-old female patient, studying on the fourth grade of elementary school, and right-handed, who underwent left hemispherectomy in a neurological rehabilitation program. Her seizures began at 10 years of age and were unresponsive to drug treatment, with functional repercussions. She underwent hemispherectomy in March 2019, with 7 years of ongoing disease, and was admitted for the rehabilitation program with a multidisciplinary team in June 2020. The quality-of-life questionnaire (WHOQOL-BREF) was applied as a comparison measure before and after the rehabilitation program, along with measures of participation on the program activities. Despite the patient’s short time in a rehabilitation program, data show the importance of an interdisciplinary intervention through the establishment of specific and contextualized objectives in chronic patients.

## RESUMO

A encefalite de Rasmussen representa uma doença rara, que consiste em uma reação inflamatória dos tecidos cerebrais, de modo crônico e lentamente progressivo. O objetivo deste estudo foi descrever o caso de uma paciente, submetida à hemisferectomia à esquerda, em março de 2019, em programa de reabilitação neurológica. Paciente do sexo feminino, 18 anos de idade, 4ª série do ensino fundamental, destra, que participou de programa de reabilitação neurológica interdisciplinar após hemisferectomia. Início das crises convulsivas aos 10 anos de idade, refratárias ao tratamento medicamentoso, com repercussão funcional. Foi submetida à hemisferectomia em março de 2019, com sete anos de doença em curso. Foi internada para programa de reabilitação, com equipe multiprofissional, em junho de 2020. Aplicou-se o questionário de qualidade de vida (WHOQOL-BREF), como medida de comparação antes e após o programa de reabilitação, além de medidas de participação nas atividades do programa. Os dados, apesar do pouco tempo da paciente em programa de reabilitação, mostram a importância da intervenção interdisciplinar, por meio do estabelecimento de objetivos realísticos e contextualizados.

## INTRODUCTION

Rasmussen encephalitis (RE) is a rare disease consisting of a chronic, slowly progressive hemispheric unilateral inflammation of the cerebral cortex, probably driven mostly by a T-cell response to one or more antigenic epitopes.^1^ ^-^ ^3^ It is characterized by difficult-to-treat focal epileptic seizures, progressive hemiparesis, and intellectual disability.^4^ RE patients, despite their normal development, occasionally deteriorate to a condition of mild to severe mental retardation, with persistent encephalitis and seizures.^5^

Medical management is based on seizures control. Polypharmacy with antiseizure medication is frequently used once seizures may be intractable at onset or become so over time. Since epilepsy is driven by ongoing inflammation, either immunosuppressive or immunomodulatory treatment has been used.^6^ Hemispherectomy, the only established method to cure seizures, with success rates of 70–80%, is usually considered for patients whose brain injury produced hemiplegia or severe hemiparesis, drug-resistant seizures, and homonymous hemianopsia. Timing of surgery is often guided by the epilepsy severity, especially if the neurological decline is associated with the appearance of contralateral, independent interictal epileptic discharges.^2^

With intense postoperative rehabilitation, patients typically regain the ability to walk independently with an ankle brace and compensate their visual field cut. The major motor disability is seen on the fine motor control in the affected side hand. The cognitive and language deficits depend on the laterality of the hemisphere disconnected.^6^

Even years after surgical treatment, there is optimal brain reorganization, if the patients undergo specific rehabilitation.^7^ ^-^ ^10^

Studies involving patients with other neurological diseases, in a chronic period, show improved functionality in a rehabilitation program.^11^

A rehabilitation program seeks interventions capable of minimizing the disability caused by the deficiency and of improving the functional potential. In the chronic phase of the injury, adaptations are used to compensate for the disability and promote greater participation in the activities of daily living and social reintegration.^12^

This interdisciplinary model of rehabilitation meets the needs of patients who require care and monitoring by several specialists. It emphasizes not only the treatment of symptoms but also the prevention of complications and, in chronic patients, psychosocial adaptation and the improvement of their functionality/social participation and participation in the community, always prioritizing realistic and attainable objectives.^12^ ^,^ ^13^

Cognitive stimulation is a patient-centered intervention for people with major cognitive deficits or chronic patients, in contextualized activities and of interest to the patient, with general/wider outcomes.^14^ ^-^ ^16^

As there are no studies in the literature addressing the therapeutic effects of interdisciplinary rehabilitation in this chronic neurological condition, the objective of this study was to describe the case of a patient in an interdisciplinary neurological rehabilitation program, who underwent left hemispherectomy, focusing on engaging and stimulating individuals/increasing personal satisfaction and social participation.

## METHODS

E.B.C. is an 18-year-old female patient, studying on the fourth grade of elementary school, and right-handed. This study was approved by the local Ethics Committee. Her seizures began at 10 years of age and were unresponsive to drug treatment, with functional implications. Preoperative neuroimaging (MRI) showed atrophy of the left caudate nucleus head, as well as hydrocephalus *ex vacuo* of the lateral ventricle anterior and posterior horns.

She underwent hemispherectomy in March 2019, with 7 years of ongoing disease, and was admitted for the rehabilitation program with an interdisciplinary team in June 2020.

Her neurological admission examination showed difficulty in understanding complex verbal commands, with good understanding of oral language. She could not speak. She had right hemianopsia and right spastic hemiparesis.

The following instruments were used for the motor assessment: the Modified Ashworth Scale, to assess muscle tone, and the Berg Balance Scale, to assess balance.

The cognitive assessment was contextualized and ecological, due to language impairment. The interview with the patient’s mother and the observation of the patient were important sources of information to design the rehabilitation program.

The rehabilitation plan was established, with objectives outlined, negotiated with the patient’s mother, which were reviewed weekly through activity evaluation groups (conducted by two professionals) and team meetings.

The contextualized neuropsychological assessment was explained as follows:

- Attentional processes impairment.
- Apathy.
- Low emotional reactivity.
- Slow information processing.
- Difficulty of the patient’s mother to deal with the patient on a daily basis.

The interdisciplinary rehabilitation plan sought the following:

- To improve attention for the patient to be able to focus and maintain the attention.
- To encourage and reinforce spontaneous attitudes and initiative taking on a daily basis, that is, to request objects, photographs, food/water, and other needs.
- To make use of the JABtalk application for supplementary communication.
- To expand interpersonal contact and socialization with professionals and other patients.
- To insert meaningful activities, such as recording videos on social networks and participating in activities involving music.
- To establish a routine of activities, seeking a higher level of participation in them.
- To provide guidance to the mother, individually and in a group, to help her understand the difficulties, redefine expectations, and for emotional support.

The quality-of-life questionnaire (WHOQOL-BREF) was applied as a comparison measure before and after the rehabilitation program. This instrument consists of four domains: physical, psychological, and social relations, and the environment.^17^ Despite being self-administered, we opted for a direct interview, due to the possibility of interpretation errors. Due to the communication impairment, the questionnaire was applied to the patient’s mother (interview mode), a person in daily contact with the patient and who was able to respond appropriately to the patient’s questions.

The quality-of-life assessment has an increasing importance in the scientific literature with not only the aspects related to the disease being valued but also the patient’s perspective in relation to her life. This concept emphasizes the subjective assessment of the quality of life and the patient’s perception of her physical, emotional, and social status. This perspective offers elements for understanding the desires, motivations, resources, and opportunities available for a person’s well-being and satisfaction, and their achievements in different domains.^18^

## RESULTS

The rehabilitation program was started on June 30, 2020, and the patient was accompanied by her mother (3 months). On admission, significant apathy was observed. E.B.C. was unable to react to environment stimuli, with social and affective withdrawal, and showed reduced motivation and behaviors aimed at goals.

The patient participated in interdisciplinary hospital rehabilitation. An interdisciplinary team was involved. Joint weekly meetings were held to discuss her progress and to review program strategies/goals. Her mother, the primary caregiver, was actively involved in the goal-setting process.

E.B.C. was referred to physical and cognitive activities, handicraft, and body perception workshops. The visits for cognitive stimulation were focused on performing attentional, visual-perceptual, and planning/sequencing activities, as well as on promoting the modulation of emotions (responsiveness to positive and negative stimuli).

She remains participative and with good attention to verbal orders, promptly responding to simple commands. When stimulated, she verbalizes, emitting mainly isolated words, but with the production of some small automatic sentences (e.g., “I don’t want”).

Regarding the motor aspects, she performed gait exercises with maximum assistance from third parties, using an unarticulated ankle orthosis on the right foot, with the ankle in a neutral position. Phenol was applied to the right tibial nerve and botulinum toxin to the right gastrocnemius and right posterior tibial muscles, guided by electrostimulation. There was an improvement in the inversion pattern, favoring the use of orthosis, with gain in gait performance with the aid of a walker and assistance from third parties, using a knee–ankle–foot orthosis for better alignment of the right lower limb.

E.B.C. was on topiramate 150 mg, keppra 750 mg, and carbamazepine 200 mg twice a day, and sertraline 100 mg.

In the activities of daily living, she was more participative. She made videos on social media and interacted with her family members. According to her mother, “she was going back to being a playful and lively person again, as she was before” [sic].

An improvement in the ability to emit words, as well as learning from task repetition, was observed. E.B.C. showed greater interaction and emotional reactivity. This case illustrates the benefits of activities and contextualized assessment, without standardized/objective instruments in patients with chronic injury and with significant communication impairment. The organization of the routine, with activities of interest and possibility to be performed, enabled greater involvement and cognitive stimulation, making the patient more adapted to her environment. For that, there was a need for her mother’s involvement in this whole process, being a facilitator in the rehabilitation.

E.B.C. was discharged after 3 months, with functional gain in physical, cognitive, and social aspects and showing improvement in the basic and instrumental activities of daily living. However, she had residual neurocognitive impairment, mainly in language, which routinely required supervision and mediation.

Table 1 shows the result before and after rehabilitation in motor measurements. Objective gain was small, but the response was better observed in functionality, assessed through observational analysis.

**Table 1.** Motor assessment before and after 3 months of rehabilitation.

Motor scales	Baseline	Post-rehabilitation
Berg Balance	4 (able to safely sit without support for 2 min)	4 (able to safely sit without support for 2 min)
Sitting balance	0 (changes from sitting to standing with moderate assistance to get up)	1 (changes from sitting to standing with minimal assistance to get up)
Change of posture	0 (change from standing to sitting with assistance from others)	0 (change from standing to sitting with assistance from others)
Orthostatic balance	0 (unable to stand for 30 s without assistance)	0 (unable to stand for 30 s without assistance)
Transfers	1 (requires assistance from 1 person to make transfers)	1 (requires assistance from 1 person to make transfers)
Total	5	6
Modified Ashworth		
Right shoulder adductors	Grade 2 elastic hypertonia	Grade 2 elastic hypertonia
Right elbow flexors	Grade 2 elastic hypertonia	Grade 2 elastic hypertonia
Right elbow extensors	Grade 2 elastic hypertonia	Grade 2 elastic hypertonia
Wrist flexors	Grade 2 elastic hypertonia	Grade 2 elastic hypertonia
Right knee extensors	Grade 2 elastic hypertonia	Grade 2 elastic hypertonia
Ankle plantar flexors	Grade 2 elastic hypertonia	Grade 2 elastic hypertonia

Following orthopedic interventions and proper orthotization, the patient progressed to functional walking training using an adapted walker, allowing for functional home displacements with supervision. Thus, adaptations are necessary in the chronic phase of the injury, aiming to minimize disabilities and enhance functionality.

Table 2 shows the patient’s improvement, through observational analysis, in the rehabilitation program activities and in the aspects of communication, body perception, cognition, activities of daily living, and behavior. The patient improved her ability of initiative and emotional engagement with other patients and professionals, and of expressing her emotions and wishes more easily, focusing her attention on the tasks performed, with less episodes of dispersion and fatigue.

**Table 2.** Description of rehabilitation program activities.

Activities	Baseline	Post-rehabilitation (3 months)
Games (connector toys, story sequence, puzzle assembly, and Lince board game)	3	1
Communication:		
Gestures	4	1
Name objects	4	1
Association of words to pictures	4	1
Word formation with moving letters	4	1
Body perception	4	2
Activities of daily living	3	2
Social interaction	4	2
Initiative	4	1
Emotional engagement	4	1

1: needs little mediation; 2: needs medium mediation; 3: needs a lot of mediation; 4: not able.

The score on the WHOQOL-BREF questionnaire showed improvement in the quality of life, when compared with the baseline assessment score, before the rehabilitation program (Table 3).

**Table 3.** Description of quality-of-life scores (before and after the rehabilitation program).

WHOQOL-BREF	Baseline score	Post-rehabilitation score (3 months)
General questions		
Quality of life	2	3
Health	3	4
Domains		
Physical	11	14
Psychological	10	16
Social relations	7	8
Environment	24	26
Total	52	64

WHOQOL-BREF: quality-of-life questionnaire.

## DISCUSSION

Although there are many publications focusing on the presentation, diagnosis, and acute treatment of RE,^2^ ^,^ ^3^ ^,^ ^19^ there is little information on the rehabilitation strategies and results.^20^ ^,^ ^21^

Changes in the behavior/mood, found in this neurological condition, are a common form of presentation^22^ and were treated with the judicious use of medications and environmental changes.

In this case report, we can observe the importance of ecological cognitive stimulation, associated with motor/physical therapy, mainly in patients who can neither undergo the application of standardized tests nor participate in the structured activities. The procedures adopted had objective, individualized goals to promote functional improvement and psychosocial adaptation.^23^

Cognitive improvement depends on the duration of epilepsy before surgery, the control of seizures after surgery, and the preservation of the contralateral hemisphere functionality.^24^ In the case described, adequate control of epilepsy and brain magnetic resonance with no alterations in the contralateral hemisphere were factors facilitating neuroplasticity, even though the surgical procedure was performed after the age range for language development.

Regarding the motor aspects, there were gains in the performance of gait exercise, through systematic training, associated with the application of phenol and botulinum toxin, as well as orthosis.

The initial assessment not only covers the complaints and difficulties presented but also explores the patient’s skills and the possibility of family support.^25^ Currently, the patient shows improved cognitive attitude and demonstrates curiosity and involvement with stimuli in the environment, as well as emotional reactivity. It should be noted that objective measures, in these cases, are not sufficient to reliably predict the impact on daily activities. It is important to assess how a given task is performed and what the gains from mediation and adaptation strategies are.

Despite the short duration of the patient in a rehabilitation program, data show the importance of an interdisciplinary intervention through the establishment of specific and contextualized objectives.

The interdisciplinary rehabilitation model shows the importance of teamwork in an integrated way, sharing information to establish broad and common goals, according to the needs and potentials of patients. These objectives will be related to a concrete activity, decided with the patient and the family. Family participation is a key factor in the outcome of the intervention, which was observed in this case report. More functional and contextualized stimulation is emphasized in tasks that were part of the daily life of the patient, that is, which brought meaning to her.

The relevance/uniqueness of the case and the challenges of the rehabilitation process are highlighted. Further studies, such as case series, could increase the level of evidence of these results.
